# Identification of ATP synthase beta subunit (ATPB) on the cell surface as a non-small cell lung cancer (NSCLC) associated antigen

**DOI:** 10.1186/1471-2407-9-16

**Published:** 2009-01-14

**Authors:** Ze-jun Lu, Qi-fang Song, Sa-sa Jiang, Qi Song, Wei Wang, Gao-hua Zhang, Bin Kan, Lan-tu Gou, Li-juan Chen, Feng Luo, Zhi Yong Qian, Jin-liang Yang, Yu Quan Wei

**Affiliations:** 1 Cancer Center and State Key Laboratory of Biotherapy, West China Hospital, West China Medical School, Sichuan University, 37 Guoxue Xiang Street, Chengdu 610041, Sichuan, PR China; 2 College of Life Science and Technology, Jinan Uuniversity, Guangzhou 510632, PR China; 3 West China Maternal and Children Hospital, Sichuan University, 37 Guoxue Xiang Street, Chengdu 610041, Sichuan, PR China

## Abstract

**Background:**

Antibody-based immuneotherapy has achieved some success for cancer. But the main problem is that only a few tumor-associated antigens or therapeutic targets have been known to us so far. It is essential to identify more immunogenic antigens (especially cellular membrane markers) for tumor diagnosis and therapy.

**Methods:**

The membrane proteins of lung adenocarcinoma cell line A549 were used to immunize the BALB/c mice. A monoclonal antibody 4E7 (McAb4E7) was produced with hybridoma technique. MTT cell proliferation assay was carried out to evaluate the inhibitory effect of McAb4E7 on A549 cells. Flow cytometric assay, immunohistochemistry, western blot and proteomic technologies based on 2-DE and mass spectrometry were employed to detect and identify the corresponding antigen of McAb4E7.

**Results:**

The monoclonal antibody 4E7 (McAb4E7) specific against A549 cells was produced, which exhibited inhibitory effect on the proliferation of A549 cells. By the proteomic technologies, we identified that ATP synthase beta subunit (ATPB) was the corresponding antigen of McAb4E7. Then, flow cytometric analysis demonstrated the localization of the targeting antigen of McAb4E7 was on the A549 cells surface. Furthermore, immunohistochemstry showed that the antigen of McAb4E7 mainly aberrantly expressed in tumor cellular membrane in non-small cell lung cancer (NSCLC), but not in small cell lung cancer (SCLC). The rate of ectopic expressed ATPB in the cellular membrane in lung adenocarcinoma, squamous carcinoma and their adjacent nontumourous lung tissues was 71.88%, 66.67% and 25.81% respectively.

**Conclusion:**

In the present study, we identified that the ectopic ATPB in tumor cellular membrane was the non-small cell lung cancer (NSCLC) associated antigen. ATPB may be a potential biomarker and therapeutic target for the immunotherapy of NSCLC.

## Background

Lung cancer is the leading cause of cancer related deaths in the world. Non-small cell lung cancer (NSCLC) accounts for more than 85% of all cases of lung cancer, and most patients with NSCLC have advanced disease at diagnosis [1,2]. The therapies for lung cancer are mainly based on traditional modes such as operation, chemotherapy and radiotherapy, however, the curative effect obtained is less satisfactory. Recently, immunotherapy for cancer has became the forth device following the traditional therapy. Antibody-based immunotherapy targeting to tumor antigen or cell surface markers has achieved some success for cancer including NSCLC, such as cetuximab, panitumumab, matuzumab and trastuzumab [3-9]. But only a few tumor-associated antigens or therapeutic targets are available at present. Identifying novel antigens (especially cellular membrane markers) will further improve tumor immunotherapy.

In the past several years, considerable progress has been made in the identification of tumor-associated antigens recognized by monoclonal antibodies (mAbs) or autoantibodies from the patients. The strategies such as serological analysis of recombinant cDNA expression libraries (SEREX), phage antibody library and ribosome display have been used to screen and identify tumor antigens. Besides, hybridoma technology can be taken as an available tool to produce anti-tumor antibodies and identify novel tumor antigens [10]. More than 1,000 tumor-associated antigens have been reported so far. The importance of these tumor antigens lies in their diagnostic and potential therapeutic utility. Moreover, those tumor antigens can also provide some prognostic information for the cancer patients.

In the present study, we produced a monoclonal antibody 4E7 (McAb4E7) specific against human lung adencarcinoma A549 cell line, which could inhibit proliferation of A549 cells. Then, by proteomic technologies, we identified ATP synthase beta subunit (ATPB) to be the corresponding antigen of McAb4E7. Immunohistochemstry showed that the antigen of McAb4E7 mainly aberrantly expressed in tumor cellular membrane in non-small cell lung cancer (NSCLC), but not in small cell lung cancer (SCLC). Our results suggested that abnormally expressed ATPB on cell surface might be a potential tumor associated antigen in the immunodiagnostics and immunotherapy for NSCLC.

## Methods

### Materials

Human lung adencarcinoma cell line A549, human small cell lung cancer cell line H-128 and human lung diploid cell line MRC-5 were bought from American Type Culture Collection (ATCC). Cells above were cultured in DMEM or RPMI1640 (Gibco) medium, with 1% penicillin-streptomycin and 10% fetal calf serum at 37°C in a 5% CO_2_-humidified atmosphere. Pinpoint cell surface protein isolation kit (Pierce Biotechnology, USA). Immobiline Dry-Strips (17 cm, PI 3–10 NL), IPG buffer, Dry-Strip cover fluid, urea, thiourea, ammonium bicarbonate were purchased from BioRad (Hercules, CA, USA). DTT, TFA, CAN, iodoacetamide, CHAPS, glycerol, agarose, ammonium persulfate, glycine, acrylamide, Bis, TEMED, SDS, Tris base, MTT, DMSO and CBB R-250 were obtained from Sigma Chemical (St. Louis, MO, USA). Other chemicals were domestic products.

### Production of McAb4E7

Membrane proteins of A549 cells were isolated with the pinpoint cell surface protein isolation kit according to the manual instructions. Six to eight weeks old female BALB/c mice were immunized each with 100 μg membrane proteins of A549 cells mixed with Freund's complete adjuvant (Sigma Chemical Co, St. Louis, Mo.) subcutaneously, and boosted each with 100 μg proteins mixed with Freund's incomplete adjuvant after 14, 21, 28 days respectively. The valences of antibodies in peripheral blood were determined by ELISA. Three days after the last boosting, sensitized spleen cells (5 × 10^8 ^cells) were harvested, mixed with 1 × 10^8 ^NS-1 myeloma cells, and fused in 50% polyethylene glycol 1500 with the proportion of 8:1. The fused cells were distributed in 96-well plates (6 × 10^5^/well) and cultured for two weeks in RPMI 1640 with 10% fetal calf serum containing hypoxanthine, aminopterin, and thymidine to select positive hybrid cells. The positive hybridoma cells were subcloned by limiting dilution. Then 10–12 weeks old female BALB/c mouse was inoculated with 3 × 10^6 ^hybridoma cells. The antibodies were further purified from the ascites via Protein-A affinity chromatography. One of the antibodies with high valence against A549 cells was named as McAb4E7. The antibody-subclass of McAb4E7 was determined by ELISA method.

### MTT cell proliferation assay

A549 cells were seeded in 96-well plate at 6,000 cells per well. 10 μg/mL and 80 μg/mL of mouse control IgG and McAb4E7 antibody were added respectively. After 24 h, 48 h and 72 h, 20 μL MTT (3-(4,5-dimethylthiazol-2-yl)-2,5-diphenyltetrazolium bromide) solution (5 mg/ml) was added to each well and incubated at 37°C for a further 4 h. Then 200 μL of dimethylsulfoxide (DMSO) was added to each well after the medium was removed. The optical density (OD) values were measured at 490 nm on a scanning multi-well spectrophotometer (BioRad Model 550, USA). Compared with the control group, the relative survival rate of remained cells at each antibody concentration was calculated from the absorbance values. The results were analyzed by ANOVA and Student-Newman-Keuls tests, the value of statistical significance being set at the p < 0.05 level.

### Western blot

The cells were cracked by the RIPA lysis buffer (50 mM Tris-HCl (pH 7.4), 1%NP-40, 0.25%Na-deoxycholate, 150 mM NaCl, 1 mM EDTA, 1 mM PMSF, 1 mg/ml Aprotinin, 1 mM Na_3_VO_4_, 1 mM NaF). Then proteins were suspended in Lammli sample buffer and centrifuged at 15, 000 rpm for 30 min. The supernatant was collected. Ten μg of each protein sample was loaded per well and separated with 12.5% SDS-PAGE. The proteins in gel were electroblotted onto PVDF membranes (Millipore) by wet blotting. After incubation in blocking buffer (1 × TBS, 0.1% Tween-20, and 5% w/v dry nonfat milk) for 1 h at room temperature, membranes were incubated in a 1:1000 dilution of McAb4E7. Then the membrane was incubated in a 1:10000 dilution of a HRP-conjugated goat-anti-mouse IgG and a HRP-conjugated anti-GAPDH antibody to confirm equal protein loading in each lane (Zhongshan Biological, Beijing, China) for 45 min at room temperature. Band containing targeting antigen was detected using an ECL detection system (Western Lighting™, PerkinElmer Life Science, Boston, MA) and exposed to X-ray film.

For 2-DE/Western blot, immediately after the second dimension run, proteins in the gel were transferred to PVDF membrane according to the method as mentioned above. The PVDF membrane was stained with coomassie R-250 after transferring, then was destained with several changes of 40% methanol, 10% acetic acid before incubated with McAb4E7.

### Flow cytometric assay

The cells were collected and washed twice with phosphate buffered saline (PBS). Then, the cells were adjusted to a concentration of 1 × 10^6 ^cells per mL, and incubated with McAb4E7 for 30 min at 4°C at 1:300 dilution. After washed three times with PBS, fluorescein-isothiocyanate (FITC)-labeled goat anti-mouse IgG (Becton Dickinson) in PBS was added and further incubated for 20 min at 4°C. Finally, the cells were washed again with PBS, and the membrane antigen expression was analyzed with a fluorescence-activated cell sorter (ESP Elite, Beckman Coulter).

### Immunohistochemistry

NSCLC tissues (n = 65), their adjacent nontumourous tissues (n = 62) and SCLC tissues (n = 10) were obtained from department of pathology in West China Hospital of Sichuan University. NSCLC tissues included 32 lung adenocarcinoma samples and 33 squamous carcinoma samples. Tissue sections were treated with 0.3% hydrogen peroxidase for 5 min, followed by blocking for 30 min with normal goat serum at room temperature. Antibody 4E7(1:1000) were applied to the blocked sections and incubated overnight at 4°C. The sections were incubated for 30 min at 37°C with HRP-labeled goat anti-mouse IgG antibody(1:500), and positive signals were visualized by development in diaminobenzidine tetrahydrochloride (DAB) solution. The sections were viewed under an Olympus Ax-70 DMC Ie CCD camera to a PC monitor. The difference between groups was analyzed by Chi-square test, and the value of statistical significance was set at the p < 0.05 level.

### Two-demensional electrophoresis (2-DE)

2 × 10^7 ^A549 cells were solubilized in 1 ml lysis solution (8 M urea, 4% CHAPS, 2 mmol/L TBP, 0.2% ampholyte, traces of bromophenol blue) at 4°C for 20 min. Insoluble material was removed by centrifugation at 15000 rpm at 4°C for 30 min. Protein concentration was determined by the method of Bradford. Samples were frozen at -70°C, and thawed immediately before use. For 17 cm IPG Ready Strips, 1 mg proteins were loaded. After rehydrating the strips for 14 h, IEF was carried out for 1 h at 200 V, 1 h at 500 V and 1 h at 1000 V; then a gradient was applied from 1000 to 8000 for 1 h and finally at 8000 V for 8 h to reach a total of 72 KVh at 20°C. Following IEF separation, gel strips were incubated for 15 min in equilibration buffer (50 mM Tris-HCl, pH 8.8, 6 M urea, 30% glycerol, 2% SDS) with 10 mg/mL DTT, followed by 15 min in equilibration buffer with 25 mg/mL iodoacetamide. Then the strips were loaded on 12.5% SDS-PAGE gel, and were electrophoresised for 20 min at a constant current of 10 mA and then at 30 mA per gel until the bromophenol blue reached the bottom of gels. Sebsquently, the gels were stained with with coomassie R-250, and destained with 40% methanol, 10% acetic acid.

### MALDI-TOF-MS/MS analysis and database search

The excised gel piece was destained at room temperature in 50 mM NH_4_HCO_3 _buffer, pH 8.8, containing 50% ACN for 1–2 h, and dehydrated with 100% ACN. The gel piece was rehydrated in 10 μL trypsin solution (50 mM NH_4_HCO_3_, pH 8.0, containing 12.5 μg/mL) for 1 h, followed by addition of 10 μL 50 mM NH_4_HCO_3 _buffer to the gel piece. After incubation at 37°C overnight, 0.5 μL incubation buffer was mixed with 0.5 μL matrix solution (CHCA, 2 mg/mL in 50% ACN, and 0.5% TFA) and pipetted directly onto the stainless steel sample plate of the mass spectrometer. The sample was analyzed by Q-TOF Premier Mass Spectrometer (Waters Micromass, Milford, MA, USA). Ionization was achieved by a nitrogen laser (337 nm) and acquisitions were performed in a V mode. Standard calibration peptides were applied to the MALDI plate as external calibration of the instrument and internal calibration using either trypsin autolysis ions or matrix was applied post acquisition for accurate mass determination. These parent ions in the mass range from 700 to 3000 m/z were selected to produce MS/MS ion spectra by CID. The collision voltage varied between 34 and 161 eV depending on the mass of the precursor ion. The MS and MS/MS data were acquired and processed using MassLynx 4.1 software (Waters Micromass, Milford, MA, USA). In a MALDI Survey scan, only one MS scan was performed. PKL files were analyzed with a licensed copy of the MASCOT 2.0 program (MatrixScience Ltd, London) against Swiss-Prot protein database with a peptide tolerance of 0.5 Da. Searching parameters were set as following: enzyme, trypsin; allowance of up to one missed cleavage peptide; the peptide mass tolerance,1.0 Da and the fragment ion mass tolerance, 0.3 Da; fixed modification parameter, carbamoylmethylation; variable modification parameters, oxidation; auto hits allowed; results format as peptide summary report. Proteins were identified on the basis of two or more peptides whose ions scores both exceeded the threshold, p < 0.05, which indicates identification at the 95% confidence level for those matched peptides.

## Results

### Production and characterization of McAb4E7

**Figure 1:**
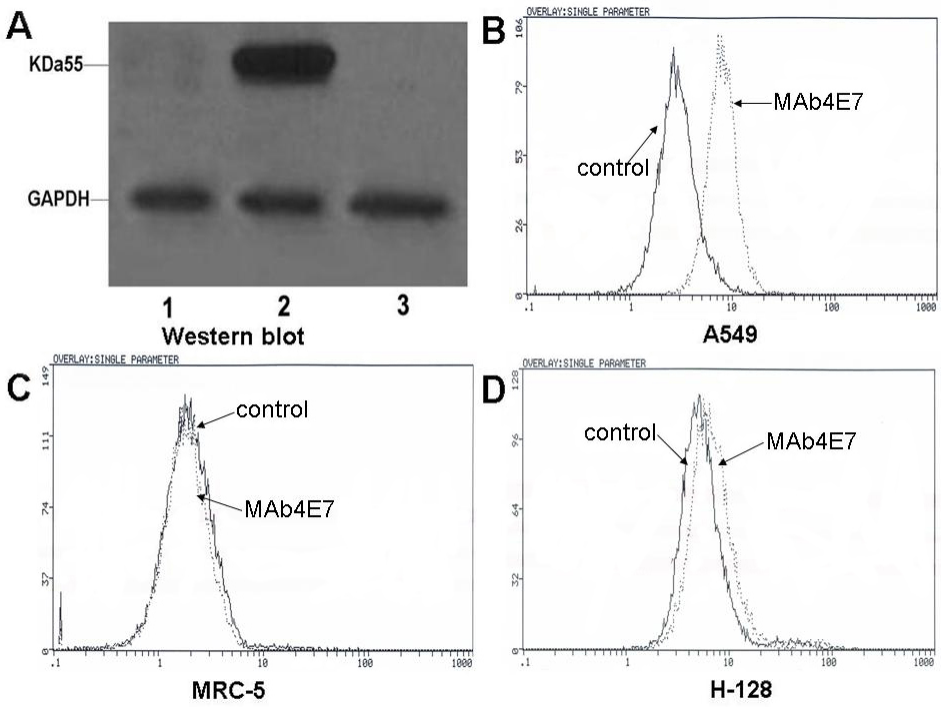
**Western bolt and flow cytometric analysis for the antigen of McAb4E7**. (A) Western bolt detection for the expression of the antigen of McAb4E7, lane1: H-128 cells; lane 2: A549 cells; lane 3: MRC-5 cells; GAPDH was used as reference, the expression of the antigen of McAb4E7 was found in A549 cells (B-D) Flow cytometry analysis for the localization of the targeting antigen, the localization of antigen of McAb4E7 was in cellular membrane of A549 cells, but not in MRC-5 and H-128 cells.

With immunizing BALB/c mice by membrane proteins of A549 cells, fourteen monoclonal hybridoma cell lines were obtained after several fusion experiments. By Western blot analysis, we found that a monoclonal antibody (McAb4E7) produced by hybridoma cell line 4E7 was specific against A549 cells but not against H-128 cell line and MRC-5 cell line (Figure 1A). Flow cytometric analysis showed that the localization of the antigen of McAb4E7 was in cellular membrane of A549 cells (Figure 1B). The subclass of McAb4E7 was identified to be IgG1 by ELISA analysis.

### Inhibitory effect of McAb4E7 on A549 cells proliferation

**Figure 2:**
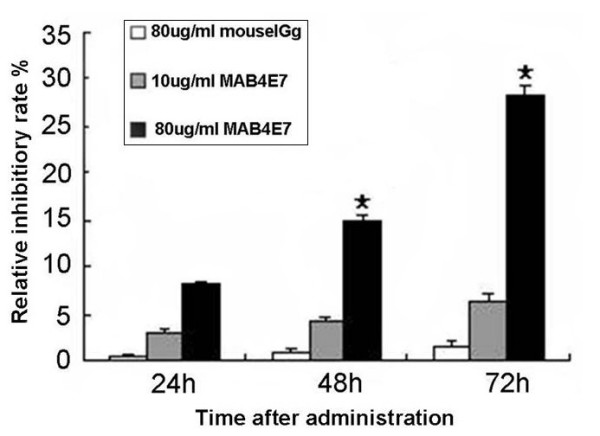
**Inhibitory effect of McAb4E7 on A549 cells**. The relative inhibitory rate of 80 μg/mL McAb4E7 on cultured A549 cells was 14.9% and 28.3% respectively after 48 h and 72 h (P < 0.05); the relative inhibitory rate of 80 μg/mL IgG antibody and 10 μg/mL McAb4E7 had no statistical difference (P > 0.05).

The effect of McAb4E7 on the proliferation of A549 cells was evaluated via MTT assay. Compared with the untreated and control IgG treated cells, the relative inhibitory rate of 80 μg/mL McAb4E7 on A549 cells was 14.9% and 28.3% respectively after 48 h and 72 h (*P *< 0.05) (Figure 2). The results indicated that McAb4E7 could inhibit the proliferation of A549 cells in vitro.

### Immunohistochemical analysis for the antigen of McAb4E7

**Figure 3:**
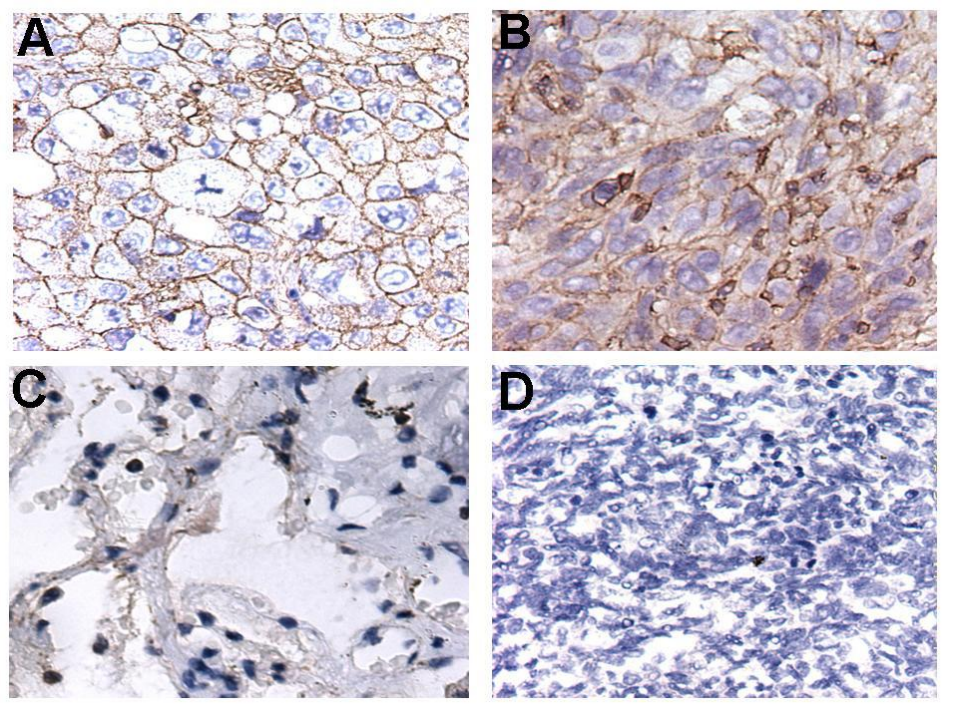
**Subcellular localization of the antigen of McAb4E7**. (A and B) The targeting antigen mainly located in the cellar membrane both in lung adenocarcinoma and squamous carcinoma (C) A little expression of the targeting antigen could be seen in the cytoplasm in the adjacent nontumourous lung tissues (D) The targeting antigen was not be found in the SCLC tissues; Magnifications: A-D × 40.

To further investigate the expression of the antigen of McAb4E7 in lung cancer tissues, we performed immunohistochemical analysis using paraffin-embedded tissue specimens. The expression of the antigen of McAb4E7 was obviously positive in the cellular membrane of lung adenocarcinoma and squamous carcinoma tissues (Figure 3A and 3B), while weak expression of the antigen of McAb4E7 could be seen in the cytoplasm in the adjacent nontumourous tissues of NSCLC (Figure 3C). The antigen of McAb4E7 was not be found in the tissues of SCLC (Figure 3D). The rate of expressed antigen of McAb4E7 in the cellular membrane in lung adenocarcinoma, squamous carcinoma and adjacent nontumourous lung tissues was 71.88%, 66.67% and 25.81% respectively (Table 1). The rate of membrane expressed antigen had significant difference between NSCLC tissues and their adjacent nontumourous tissues (P < 0.05), and it had no statistical difference between lung adenocarcinoma and squamous carcinoma (P > 0.05).

**Table 1:** Ectopic expressioned ATPB in the cellular membrane in lung cancer tissues

Pathological types	Number of samples	Ectopic localization of ATPB in the cellular membrane	Rate (%)
NSCLC Adenocarcinoma	32	23	71.88
Squamous carcinoma	33	22	66.67
Adjacent nontumourous tissues of NSCLC	62	16	25.81
SCLC	10	0	0

The rate of ectopic expressed ATPB in the cellular membrane had significant difference between NSCLC tissues and their adjacent nontumourous tissues (P < 0.05); the rate of ectopic expressed ATPB had no statistical difference between lung adenocarcinoma and squamous carcinoma (P > 0.05).

### Identification of the antigen of McAb4E7 by 2-DE/Western blot and MALDI-TOF-MS/MS analysis

**Figure 4:**
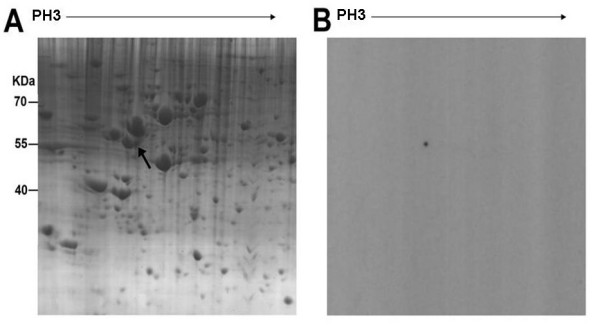
**2-DE/Western blot analysis of the targeting antigen of McAb4E7**. (A) The quarter 2-DE map of A549 cells, black arrow indicated the targeting antigen (B) Western blot detection for the targeting protein spot recognized by McAb4E7.

To recognize the targeting antigen of McAb4E7, 2-DE/Western blot was performed with A549 cells. Preliminary experiment with Immobiline Dry-Strips (7 cm, 3–10 NL) indicated that the positive protein spot was located at *PI *5 and *MW *55 kDa in the gel. To improve the resolution, 17 cm gels (17 cm, 3–10 NL) were used, and a quarter of the gel was subjected to Western blot in view of the *PI *and *MW *of the corresponding antigen of McAb4E7 (Figure 4). Then, the protein spot, corresponding to those showing a positive reaction with the antibody 4E7 in 2-DE/Western blot, was excised from the gel (Figure 4A).

**Figure 5:**
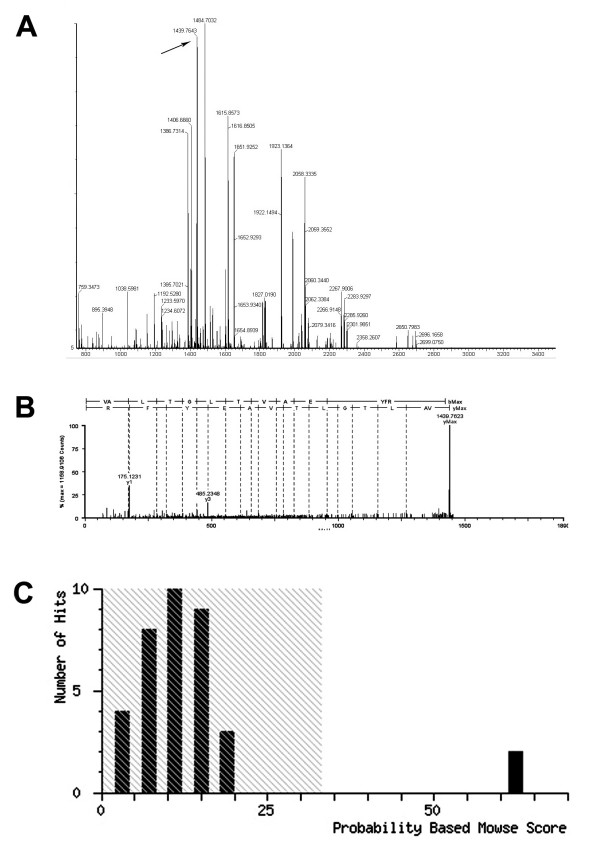
**MALDI-TOF-MS and MS/MS analysis of the antigen of McAb4E7**. (A) Peptide mass fingerprinting (PMF) of the targeting protein recognized by McAb4E7. Black arrow in the image indicated the peak of ATPB (B) MS/MS spectrum of peak [M +2H]^2+ ^at m/z 1439.77 from positive spot matched sequence RVALTGLTVAEYFRD of ATPB (C) Probability based mowse score.

The excised gel piece was destained and trypsinized into peptides for MS and MS/MS analysis. Mass spectra were acquired with a Q-TOF Premier Mass Spectrometer (Figure 5). The MS/MS data, including the mass values, the intensity and the charge of the precursor ions, were analyzed with a licensed copy of the Mascot 2.0 program against SWISS-PROT protein database. The charged ion of 1439.76 m/z and its fragment ions revealed the peptide sequence, R.VALTGLTVAEYFR.D, unique to ATPB (Figure 5A and 5B). The protein spot was identified as ATP synthase beta subunit (ATPB). The score was 62 (*P *< 0.05) (Figure 5C).

## Discussion

In this study, we produced the monoclonal antibody 4E7 using membrane proteins of lung adenocarcinoma cells A549 as the immunogen by hybridoma technology, and demonstrated that this antibody could inhibit the proliferation of A549 cells. Most importantly, we identified the ectopic ATP synthase beta subunit (ATPB) in the cellular membrane to be the target antigen of McAb4E7, which indicated that ATPB might be a potential target for NSCLC immunotherapy.

There are several strategies for the identification of tumor-associated antigens, such as serological analysis of recombinant cDNA expression libraries (SEREX), phage antibody library, ribosome display and tumor special antibody cloning. We performed the last strategy here. Generally speaking, potential molecular targets for mAb-mediated immunotherapy must be accessible to the antibodies, it is to say that the antigens should be cell surface proteins or soluble growth factors. For this reason, we used the membrane proteins of A549 cells as the immunogen in our hybridoma strategy. As expected, the antibody secreted from hybridoma clone 4E7, specifically recognized the cell surface protein in A549 cells and NSCLC tissues was obtained.

ATPB is a subunit of ATP synthases constitutively expressed in the inner mitochondrial membrane in normal cells [11]. However, some researches have reported the ectopic expression of ATP synthases in primary cultured endothelial cells [12-16]. Colon carcinoma cells and prostate carcinoma cells also expressed ATP synthase on the cell surface [17-20]. In present study, we found that the expression of ATPB was also ectopically located on the cell surface and up-regulated in human lung adencarcinoma cell line A549, as well as in NSCLC tissues. The rates of ectopic expressed ATPB in the cellular membrane in lung adenocarcinoma and squamous carcinoma were significantly higher than their adjacent nontumourous lung tissues. These findings indicated that the ectopic expression of ATPB would be a tumor-associated antigen of NSCLC.

It was reported that angiostatin, kringle 1–5 (K1–5) of plasminogen, McAb against ATPB and small molecular inhibitors could bind to ATP synthase on the cell surface, and inhibited endothelial cell proliferation, migration, even triggered apoptosis [21-23]. In the present study, we found that McAb4E7 also exhibited inhibitory effect on the proliferation of A549 cells to some extent. ATP synthase on the cell surface was more active at low extracellular pH, such as in the tumor microenvironment. So ectopic ATPB might play important role in the tumor growth and development, and would be a potential target for NSCLC immunotherapy. However, the functions of ATPB and McAb4E7 need to be further studied.

Currently, proteomic technologies have been implemented in analysis for cells and tissues protein expression on large scale [24]. After failing to capture of the antigen of McAb4E7 with immunoprecipitate technology, we employed 2-DE/Western blot approach to locate and recognize the positive protein spot responsed to McAb4E7. Ultimately, ATPB was identified to be the corresponding antigen of McAb4E7. So the proteomic technologies provide us a potent tool to identify tumor associated antigens, especially for identifying cellular membrane antigens [25,26].

## Conclusion

In the present study, we identified ectopic localized ATPB as the corresponding antigen of McAb4E7 with the proteomic technologies. ATPB on the cell surface may be a potential biomarker and therapeutic targets for the immunotherapy of NSCLC. In order to explore the function of ectopic localized ATPB and McAb4E7, more work need to be done to evaluate their clinical applicability and specificity in ongoing study. Furthermore, the marriage of target identification with antibody enhancement technologies will ultimately be translated into new and improved therapies for cancer patients.

## Competing interests

The authors declare that they have no competing interests.

## Authors' contributions

ZJL and QFS carried out the 2-DE/western-blot and drafted the manuscript. QS and SSJ performed the flow cytometric assay. WW, BK and GHZ carried out the immunohistochemical and western-blot, as well as the statistical analysis. LTG and LJC performed MALDI-TOF MS studies. ZYQ helped in drafting the manuscript. JLY, FL and YQW participated in the design of the study. Among them, JLY critically revised the manuscript and FL contributed to the supply of lung cancer tissues. All authors read and approved the final manuscript.

## Pre-publication history

The pre-publication history for this paper can be accessed here:

http://www.biomedcentral.com/1471-2407/9/16/prepub
